# Prediction of Ischemic Events after Percutaneous Coronary Intervention: Thrombelastography Profiles and Factor XIIIa Activity

**DOI:** 10.1055/s-0038-1645876

**Published:** 2018-05-07

**Authors:** Rolf P. Kreutz, Glen Schmeisser, Andrea Schaffter, Sri Kanuri, Janelle Owens, Benjamin Maatman, Anjan Sinha, Elisabeth von der Lohe, Jeffrey A. Breall

**Affiliations:** 1 Krannert Institute of Cardiology, Indiana University School of Medicine, Indianapolis, Indiana, United States; 2 Department of Clinical Pharmacology, Indiana University School of Medicine, Indianapolis, Indiana, United States

**Keywords:** thrombelastography, fibrin, factor XIII, percutaneous coronary intervention, myocardial infarction

## Abstract

**Background** High plasma fibrin clot strength (MA) measured by thrombelastography (TEG) is associated with increased risk of cardiac events after percutaneous coronary interventions (PCIs). Factor XIIIa (FXIIIa) cross-links soluble fibrin, shortens clot formation time (TEG-K), and increases final clot strength (MA).

**Methods** We analyzed platelet-poor plasma from patients with previous PCI. Kaolin-activated TEG (R, K, MA) in citrate platelet-poor plasma and FXIIIa were measured ( *n* = 257). Combined primary endpoint was defined as recurrent myocardial infarction (MI) or cardiovascular death (CVD). Relationship of FXIIIa and TEG measurements on cardiac risk was explored.

**Results** FXIIIa correlated with TEG-MA ( *p* = 0.002) and inversely with TEG-K ( *p* < 0.001). High MA (≥35.35 mm; *p* = 0.001), low K (<1.15 min; *p* = 0.038), and elevated FXIIIa (≥83.51%; *p* = 0.011) were associated with increased risk of CVD or MI. Inclusion of FXIIIa activity and low TEG-K in risk scores did not improve risk prediction as compared with high TEG-MA alone.

**Conclusion** FXIIIa is associated with higher plasma TEG-MA and low TEG-K. High FXIIIa activity is associated with a modest increase in cardiovascular risk after PCI, but is less sensitive and specific than TEG-MA. Addition of FXIIIa does not provide additional risk stratification beyond risk associated with high fibrin clot strength phenotype measured by TEG.

## Introduction

Coronary arterial thrombosis is a complex pathologic cascade involving diseased endothelium, exposure of subendothelial matrix, platelet activation, platelet aggregation, and generation of thrombin ultimately leading to assembly of a shear resistant platelet–fibrin thrombus. The contributions of both platelets and fibrin to mechanical properties of clot formation have been well studied. 1 2

Increasing interest has focused on the ability to personalize medical therapy across all subspecialties including those focusing on treatment of cardiovascular disease. 3 4 Clinical trials have started to focus on treating particular subgroups of patients with coronary artery disease in secondary prevention, such as patients with multiple cardiovascular risk factors, to isolate either high-risk subgroups or groups expected to respond to the experimental therapy. 5

Thrombelastography (TEG) is an ex vivo thrombosis assay that is able to measure the kinetics of clot formation. 6 7 High clot strength measured by TEG has been found to be a marker associated with increased thrombotic risk in various clinical circumstances. 8 9 We recently described our findings of an association with elevated plasma fibrin clot strength measured by TEG and increased risk of future recurrent myocardial infarction (MI) and stent thrombosis in a cohort of patients with coronary artery disease and percutaneous coronary intervention (PCI). 10

Factor XIII (FXIII) is activated by thrombin and as the final enzymatic step in the coagulation cascade, it cross-links assembled soluble fibrin strands into a solid, shear resistant fibrin network. 2 11 12 13 In addition to cross-linking of fibrin strands, FXIIIa also has other anti-fibrinolytic functions, and participates in platelet-mediated clot contraction. 13 14 Deficiency of FXIII causes severe bleeding diathesis and restitution of FXIII in FXIII-deficient plasma dose-dependently increases clot strength measured by TEG. 11 The contribution of FXIIIa activity to the risk of recurrent coronary thrombosis in patients treated with dual-antiplatelet therapy has not been previously studied.

We intended to further investigate the relative contribution of factor FXIIIa activity on ischemic risk, as well as interaction of FXIIIa with other TEG parameters (MA: maximal clot strength; R: reaction time; K: clot formation time) in the previously published cohort. 10

## Methods

### Study Design and Patient Population

The study protocol was approved by the Indiana University Institutional Review Board. All subjects provided written informed consent. We enrolled subjects among patients referred for cardiac catheterization or in follow-up to a cardiac catheterization. Subjects were included in this analysis if they had angiographically established coronary artery disease and had undergone PCI. In addition, to be included in this analysis, all subjects had to have had both plasma TEG and FXIIIa measurements completed.

### Blood Samples

Blood samples were collected into Vacutainer tubes containing Na-citrate 3.2%. Whole citrate blood was centrifuged at 2,000 × *g* for 15 minutes and resulting platelet-poor citrate plasma was stored at −80°C until analysis. Blood samples were obtained prior to or at least 12 hours after administration of heparin or bivalirudin.

### Thrombelastography

We performed kaolin-activated TEG in citrate platelet-poor plasma according to the manufacturer's instructions (TEG5000 system, Haemonetics, Braintree, Massachusetts, United States). Citrate plasma was mixed with kaolin, inverted five times, and then loaded in a heparinase-coated cup containing 20 µL of CaCl _2_ . TEG was stopped after maximal fibrin clot strength was recorded. Time to fibrin formation or reaction time (R, min), clot formation time (defined as time from beginning of clot formation until clot firmness amplitude reaches 20 mm; K, min), and maximal clot strength (MA, mm) were recorded.

### Factor XIIIa

Factor XIIIa concentration (FXIII–subunit A) was measured by enzyme-linked immunoassay in citrate plasma according to the manufacturer's instructions (FXIIIa [human] ELISA kit; Aniara, West Chester, Ohio, United States). In the assay, the FXIIIa concentration is expressed as percentage, standardized to a normal human-citrated plasma pool. The average normal concentration of FXIII tetramer in plasma is ∼25 μg/mL according to the manufacturer of the assay (Aniara).

### Clinical Endpoints

The primary combined endpoint was defined as first occurrence of cardiovascular death (CVD) or MI. Myocardial infarction was defined according to the universal definition of MI. 15 Secondary endpoints included CVD, MI, stent thrombosis, and bleeding. Stent thrombosis was defined as definite, probable, and possible, based on the Academic Research Consortium's definition of stent thrombosis. 16 Bleeding was recorded if severe or life-threatening as defined by GUSTO criteria. 17 We evaluated clinical endpoints through review of electronic medical records, and last clinical follow-up was used as last censored time event. If available, coronary angiograms were reviewed to ascertain stent thrombotic events.

### Statistics

We employed SPSS 23.0 (IBM, United States) for statistical analysis. Significance was defined as *p* < 0.05 and all tests were conducted two-sided, with values represented as mean ± SD except as otherwise stated. Continuous normally distributed data were compared with unpaired Student's *t* -test, and categorical variables were compared using the *χ* ^2^ test. Survival analysis was performed using Kaplan–Meier method. We conducted multivariate Cox regression analysis for clinical endpoints with high MA, high MA/low K, and high FXIIIa, with forward conditional adjustment for baseline clinical variables.

## Results

A total of 257 subjects were included in the analysis. The mean age of patients was 57.2 ± 10 years. The clinical variables of subjects included in the analysis are summarized in Table 1 . Mean time of follow-up was 2.9 years. The primary endpoint of CVD and MI occurred in 14.4% and definite stent thrombosis in 3% of subjects.

**Table 1:** Baseline demographics and clinical variables for total study population, and grouped according to low and high FXIIIa

Variables	Total ( *n* = 257)	Low FXIIIa (<83.51%) ( *n* = 116)	High FXIIIa (≥83.51%) ( *n* = 141)	*p* -Value
Age (y)	57.2 ± 9.9	59.3 ± 10	55.4 ± 9	0.001
BMI (kg/m ^2^ )	31.7 ± 6.9	30.8 ± 6.6	32.3 ± 7.1	0.083
Male gender (%)	152/257 (59%)	63/116 (54%)	89/141 (59%)	0.15
African American (%)	61/257 (24%)	22/116 (19%)	39/141 (28%)	0.1
Smoking (%)	97/257 (38%)	43/116 (37%)	54/141 (38%)	0.84
Hypertension (%)	237/257 (92%)	108/116 (93%)	129/141 (92%)	0.63
Hyperlipidemia (%)	231/257 (90%)	108/116 (93%)	123/141 (87%)	0.12
Diabetes mellitus (%)	111/257 (43%)	53/116 (46%)	58/141 (41%)	0.46
History of myocardial infarction	168/257 (65%)	72/116 (62%)	96/141 (68%)	0.31
History of CABG	51/257 (20%)	25/116 (22%)	26/141 (18%)	0.53
Congestive heart failure (%)	41/257 (16%)	18/116 (16%)	23/141 (16%)	0.86
Clinical presentation				
STEMI	37/257 (14%)	11/116 (10%)	26/141 (18%)	0.042
NSTEMI	56/257 (22%)	20/116 (17%)	36/141 (26%)	0.11
Unstable angina	65/257 (25%)	34/116 (29%)	31/141 (22%)	0.18
Stable CAD	95/257 (37%)	51/116 (44%)	44/141 (31%)	0.035
PCI vessel				
Left main	3/257 (1%)	1/116 (1%)	2/141 (1%)	0.68
LAD	109/257 (42%)	50/116 (43%)	59/141 (42%)	0.84
CX	64/257 (25%)	26/116 (22%)	38/141 (27%)	0.4
RCA	113/257 (44%)	47/116 (41%)	66/141 (47%)	0.31
Number of stents implanted	1.52 ± 0.8	1.44 ± 0.8	1.59 ± 0.8	0.16
Drug eluting stents	192/257 (75%)	86/116 (74%)	106/141 (75%)	0.85
Paclitaxel eluting stent	41/257 (16%)	23/116 (20%)	18/141 (13%)	0.12
Sirolimus eluting stent	14/257 (5%)	5/116 (4%)	9/141 (6%)	0.47
Zotarolimus eluting stent	8/257 (3%)	2/116 (2%)	6/141 (4%)	0.25
Everolimus eluting stent	127/257 (49%)	53/116 (46%)	74/141 (53%)	0.28
Beta-blockers (%)	236/257 (92%)	110/116 (95%)	126/141 (89%)	0.11
ACE-inhibitors/ARB (%)	185/257 (71%)	78/116 (67%)	104/141 (74%)	0.25
Calcium channel blockers (%)	38/257 (15%)	21/116 (18%)	17/141 (12%)	0.17
Statins	226/257 (88%)	101/116 (87%)	125/141 (89%)	0.7
Aspirin	256/257 (99.6%)	115/116 (99%)	141/141 (100%)	0.27
Clopidogrel	210/257 (82%)	101/116 (87%)	109/141 (77%)	0.044
Prasugrel	34/257 (13%)	9/116 (8%)	25/141 (18%)	0.02
Ticagrelor	7/257 (3%)	2/116 (2%)	5/141 (4%)	0.37

Abbreviations: ACE, angiotensin-converting enzyme; ARB, angiotensin receptor blocker; CABG, coronary artery bypass grafting; CAD, coronary artery disease; CX, circumflex; BMI, body mass index; LAD, left anterior descending; NSTEMI, non-ST-elevation myocardial infarction; PCI, percutaneous coronary intervention; STEMI, ST-elevation myocardial infarction.

Note: Comparison of variables with *t* -test for continuous variables and chi-square for categorical variables.

**Fig. 1:**
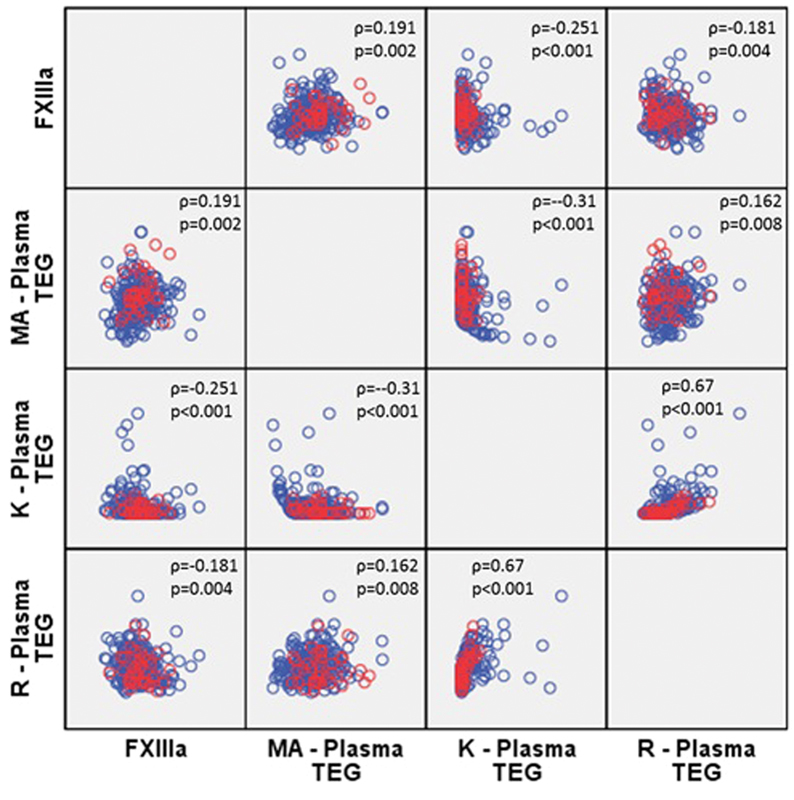
Scatterplot of thrombelastography (TEG) and FXIIIa measures in subjects without events (blue) and subjects with cardiovascular death or MI (red). FXIIIa, factor XIIIa; TEG-K, clot formation time; TEG-MA, maximal fibrin clot strength; TEG-R, time to fibrin formation.

Factor XIIIa concentration correlated with TEG-MA ( *ρ* = 0.191; *p* = 0.002) and inversely with TEG-K ( *ρ* = −0.251; *p* < 0.001), but not TEG-R ( Fig. 1 ). Clot formation time (TEG-K) was correlated with time to clot formation (TEG-R; *ρ* = 0.61; *p* < 0.001), and inversely with clot strength (TEG-MA) ( *ρ* = −0.31; *p* < 0.001). Scatterplot demonstrates clustering of recurrent ischemic events in cases with high TEG-MA, low TEG-K, and elevated FXIIIa ( Fig. 1 ). Maximal clot strength was significantly higher in subjects with occurrence of the primary endpoint during follow-up (recurrent CVD or MI; TEG-MA: 39.6 ± 7.7 vs. 35.4 ± 7.5 mm; *p* = 0.002) and clot formation time was shorter (TEG-K: 1.15 ± 0.5 vs. 1.54 ± 1.3 minutes; *p* = 0.002; Fig. 1 ), as previously published. Patients with definite stent thrombosis during follow-up had significantly higher fibrin MA than subjects without definite stent thrombosis (TEG-MA: 41.6 ± 9 vs. 35.8 ± 8 mm; *p* = 0.048). Patients with possible, probable, or definite stent thrombosis during follow-up demonstrated significantly higher MA than subjects without stent thrombosis (TEG-MA: 44.1 ± 10 vs. 35.7 ± 7 mm; *p* = 0.001). Time to clot formation (R) was not significantly different for subjects with primary endpoint (TEG-R: 6.6 ± 3 vs. 7 ± 2.9 minutes; *p* = 0.38) or definite stent thrombosis (TEG-R: 5.9 ± 2 vs. 7 ± 3 minutes; *p* = 0.37) as compared with those without events.

Optimal cutoff for high fibrin clot strength (MA ≥ 35.35 mm) was determined by receiver operating curve (ROC) analysis for the primary endpoint (area under curve [AUC] = 0.652, *p* = 0.002), as previously published. 10 To assess the added prognostic value of FXIIIa to TEG-MA measurements, we divided the study group into subjects with either low or high FXIIIa, with cutoff (<83.51%) derived from ROC analysis (AUC: 0.572, *p* = 0.164; Table 1 ). Similarly, we determined the cutoff for low TEG-K (K < 1.15 min, AUC: 0.6; *p* = 0.06) for inclusion in risk prediction score.

Subjects with high FXIIIa (≥89.51%) were younger, had more often presented with STEMI, and were more likely to have been treated with prasugrel ( Table 1 ). Subjects with high FXIIIa showed shorter clot formation times (TEG-K) and higher maximal clot strength (TEG-MA), but no significant difference in time to clot formation (TEG-R; Table 2 ).

**Table 2:** Plasma TEG measures stratified by low and high FXIIIa

	Low FXIIIa (<83.51%)	High FXIIIa (≥83.51%)	*p* -Value
TEG-R (min)	7.1 ± 2.7	6.8 ± 3.1	0.33
TEG-K (min)	1.6 ± 1.3	1.3 ± 1.1	0.041
TEG-MA (mm)	34.3 ± 7.8	37.4 ± 7.2	0.001

**Fig. 2:**
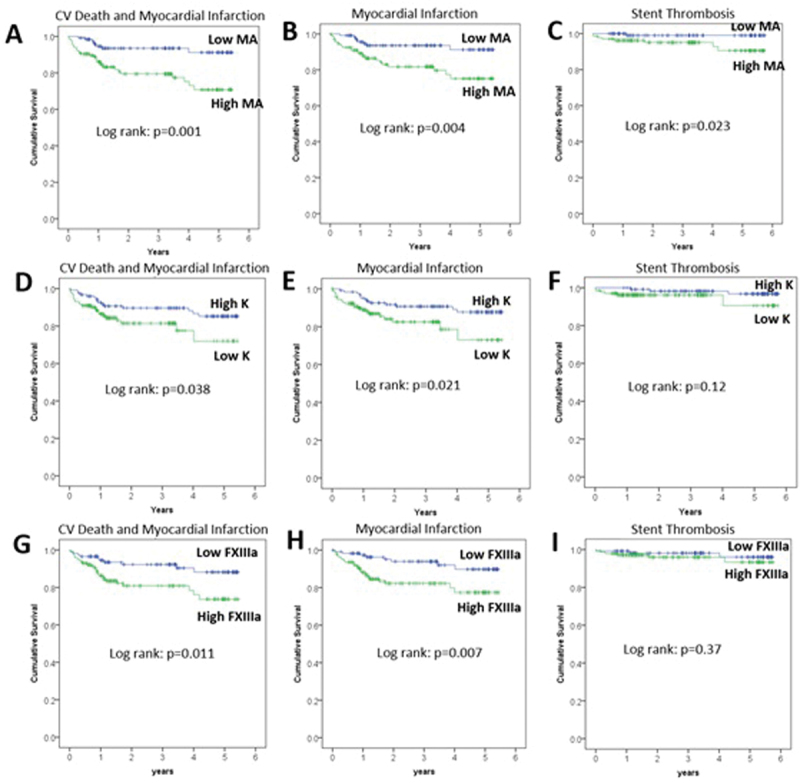
Kaplan–Meier survival analysis according to high (≥35.35 mm) versus low TEG-MA (<35.35 mm) ( **A–C** ), high (≥1.15 min) versus low TEG-K (>1.15 min) ( **D–F** ), and high (≥83.51%) versus low FXIIIa (<83.51%) ( **G–I** ) for primary endpoints of CVD or MI, MI ( **C** ), and possible/probable/definite stent thrombosis.

Survival free analysis by Kaplan–Meier demonstrated significantly increased risk for the primary combined endpoint of CVD and MI, as well as MI and stent thrombosis ( Fig. 2 ) in subjects with high clot strength (MA; Fig. 2 ), as previously reported. Short clot formation time (K) and high FXIIIa were both associated with significantly increased risk of CVD and MI, and MI alone, but not stent thrombosis ( Fig. 2 ).

Cox regression analysis was performed with forward conditional adjustment for baseline clinical variables. Both unadjusted and adjusted hazard ratios and 95% confidence intervals for individual clinical endpoints are provided in Table 3 for high versus low MA. High MA remained associated with increased hazard for the occurrence of CVD or MI, CVD, and MI after adjustment in multivariate analysis ( Table 3 ). There was a nonsignificant trend toward increased bleeding events in subjects with low MA ( Table 3 ).

**Table 3:** Unadjusted and adjusted hazard ratio for clinical outcomes associated with high and low TEG MA in Cox regression analysis

Clinical events	Low MA (<35.35 mm) ( *n* = 122)	High MA (≥35.35 mm) ( *n* = 135)	Unadjusted hazard ratio (95% CI)	*p* -Value	Adjusted hazard ratio (95% CI)	*p* -Value
Cardiovascular death or myocardial infarction	8/122 (6.6%)	28/135 (20.7%)	3.5 (1.6–7.8)	0.002	3.39 (1.5–7.2)	0.003
Cardiovascular death	0/122 (0%)	8/135 (5.8%)	64.2 (0.2–18984)	0.15	64.2 (0.2–18984)	0.15
Myocardial infarction	8/122 (6.6%)	24/135 (17.8%)	3.05 (1.4–6.8)	0.006	2.74 (1.1–6.3)	0.017
Definite, probable, or possible stent thrombosis	1/122 (0.8%)	8/135 (5.9%)	7.7 (0.96–61.3)	0.055	5.2 (0.64–42.1)	0.12
Bleeding	6/122 (4.9%)	2/135 (1.5%)	0.32 (0.07–1.6)	0.17	0.23 (0.04–1.3)	0.09

The combination of low TEG-K with high TEG-MA was associated with increased risk of occurrence of the primary endpoint ( Figs. 1 and 2 ), and therefore a “TEG risk score” was explored that would focus on a more select group of subjects at risk who exhibited both high MA and low K. Both unadjusted and adjusted hazard ratios for high TEG risk score were significant for occurrence of CVD/MI, MI, and probable, possible, and definite stent thrombosis ( Table 4 ). Next the risk associated with high FXIIIa was analyzed and increased hazard was observed for occurrence of CVD and MI, and MI alone, that remained significant after adjustment in multivariate analysis ( Table 5 ). High FXIIIa was associated with more frequent bleeding events, although this difference was not statistically significant ( Table 5 ). Next the integration of high MA, low K, and high FXIII into a combined TEG/FXIII risk score was explored. The presence of at least two of three risk variables (high MA, low K, and high FXIIIa) was associated with increased risk for CVD and MI, MI alone, and stent thrombosis after multivariate adjustment ( Table 6 ). Sensitivity and specificity of each risk score are summarized in Table 7 . Addition of low K, high FXIII, individually or in combined risk score, did not significantly improve sensitivity and specificity as compared with use of high TEG-MA alone with lower AUC values for endpoints listed ( Table 7 ).

**Table 4:** Unadjusted and adjusted hazard ratio for clinical outcomes associated with presence of combination of high MA and low K in Cox regression analysis

Clinical events	Low TEG risk score (either MA < 35.35 or K ≥ 1.15 min) ( *n* = 171)	High TEG risk score (MA ≥ 35.35 mm and K < 1.15 min) ( *n* = 86)	Unadjusted hazard ratio (95% CI)	*p* -Value	Adjusted hazard ratio (95% CI)	*p* -Value
Cardiovascular death or myocardial infarction	16/171 (9.4%)	20/86 (23.3%)	3.53 (1.8–6.9)	0.0002	3.48 (1.75–6.9)	0.0004
Cardiovascular death	4/171 (2.3%)	4/86 (4.7%)	2.8 (0.68–11.8)	0.15	3.3 (0.8–13.7)	0.1
Myocardial infarction	14/171 (8.2%)	18/86 (20.9%)	3.7 (1.8–7.5)	0.0004	3.56 (1.72–7.4)	0.001
Definite, probable, or possible stent thrombosis	3/171 (1.8%)	6/86 (7%)	6.1 (1.45 -25.3)	0.013	6.2 (1.58–24.2)	0.036
Bleeding	7/171 (5.1%)	1/86 (1.2%)	0.39 (0.05–3.2)	0.38	0.19 (0.02–2)	0.17

**Table 5:** Unadjusted and adjusted hazard ratio for clinical outcomes associated with high and low FXIIIa in Cox regression analysis

Clinical events	Low FXIIIa (<83.51) ( *n* = 116)	High FXIIIa (≥83.51%) ( *n* = 141)	Unadjusted hazard ratio (95% CI)	*p* -Value	Adjusted hazard ratio (95% CI)	*p* -Value
Cardiovascular death or myocardial infarction	10/116 (8.6%)	26/141 (18.4%)	2.5 (1.2–5.2)	0.014	2.39 (1.14–5)	0.022
Cardiovascular death	2/116 (1.7%)	6/141 (4.3%)	2.89 (0.58–14.4)	0.2	3.29 (0.58–18.7)	0.18
Myocardial infarction	8/116 (6.9%)	24/141 (17%)	2.88 (1.29–6.4)	0.01	3.04 (1.3 -7.1)	0.01
Definite, probable, or possible stent thrombosis	3/116 (2.6%)	6/141 (4.3%)	1.9 (0.47 -7.6)	0.37	1.56 (0.34 -7.2)	0.57
Bleeding	1/116 (0.9%)	7/141 (5%)	7.5 (0.9–61)	0.06	7.2 (0.87–59.1)	0.07

**Table 6:** Unadjusted and adjusted hazard ratio for clinical outcomes associated with high (>1) and low (≤1) TEG and FXIIIa risk score in Cox regression analysis

Clinical events	Low combined TEG/FXIIIa risk score (0–1) ( *n* = 110)	High combined TEG/FXIIIa risk score ( 2 3 ) ( *n* = 145)	Unadjusted hazard ratio (95% CI)	*p* -Value	Adjusted hazard ratio (95% CI)	*p* -Value
Cardiovascular death or myocardial infarction	8/110 (7.3%)	28/145 (19.3%)	3.45 (1.56–7.6)	0.002	3.6 (1.61–8)	0.002
Cardiovascular death	1/110 (0.9%)	7/145 (4.8%)	7.3 (0.88–60)	0.065	7.2 (0.86–59)	0.069
Myocardial infarction	7/110 (6.4%)	25/145 (17.2%)	3.6 (1.5–8.36)	0.003	3.73 (1.54–9)	0.004
Definite, probable, or possible stent thrombosis	1/110 (0.9%)	8/145 (5.5%)	8.1 (1.0 -65.1)	0.05	9.7 (1.08 -87.2)	0.043
Bleeding	5/110 (4.5%)	3/145 (2.1%)	0.61 (0.14–2.59)	0.5	0.38 (0.07–2)	0.26

Note: TEG risk score (number of categories present: TEG-MA ≥ 35.35 mm; TEG-K < 1.15 min; FXIIIa ≥ 83.51%).

**Table 7:** Sensitivity, specificity, PPV, NPV, and c-statistic for high TEG-MA, combination of high TEG-MA/low TEG-K, and combined TEG MA/K/FXIIIa score for CVD, MI, and possible/probable/definite ST

Score	Outcome	Sensitivity	Specificity	PPV	NPV	AUC	*p* -Value
TEG (MA ≥ 35.35 mm)	CVD/MI	77.8%	51.6%	20.7%	93.4%	0.647	0.005
TEG (MA ≥ 35.35 mm)	MI	75%	50.7%	17.8%	93.4%	0.628	0.019
TEG (MA ≥ 35.35 mm)	ST	88.9%	48.8%	5.9%	99.1%	0.688	0.055
TEG (MA ≥ 35.35 mm and K < 1.15 min)	CVD/MI	55.5%	70.1%	23.3%	90.6%	0.628	0.013
TEG (MA ≥ 35.35 mm and K < 1.15 min)	MI	56.3%	69.8%	20.9%	91.8%	0.63	0.017
TEG (MA ≥ 35.35 mm and K < 1.15 min)	ST	66.7%	67.8%	7%	98.2%	0.672	0.08
TEG and FXIIIa score (>1)	CVD/MI	78%	46.6%	19.3%	92.7%	0.624	0.017
TEG and FXIIIa score (>1)	MI	78.1%	46.2%	17.2%	93.6%	0.624	0.023
TEG and FXIIIa score (>1)	ST	88.9%	44.3%	5.5%	99.1%	0.668	0.087

Abbreviations: CVD, cardiovascular death; MI, myocardial infarction; NPV, negative predictive value; PPV, positive predictive value; ST, stent thrombosis.

## Discussion

The results of our study demonstrate that patients who form mechanically shear resistant, high strength plasma fibrin clot (high MA) with short clot formation time (low K) appear to be at risk for recurrent thrombotic events after coronary stenting, in specific CVD and MI, as well as stent thrombosis. We demonstrate a correlation between these parameters to a certain extent, particularly MA, K, and R. In addition, FXIIIa levels correlate with final maximal plasma fibrin clot strength and inversely with clot formation time, as previously demonstrated for whole blood and plasma TEG measurements in normal control populations. 2 11 Our exploratory analysis demonstrates that high FXIIIa was associated with modest increase in risk of recurrent CVD and MI, but not stent thrombosis. Inclusion of TEG-K and FXIIIa into exploratory risk scores did not further increase the performance of high plasma fibrin TEG-MA as predictor of recurrent thrombotic events after coronary stenting. While high clot strength (MA) in whole blood has been accepted as a risk variable for arterial thrombosis, there has been less awareness of clot formation time (K) as additional parameter of clot kinetics in prognostic risk, and our findings of ischemic risk associated with this parameter are noteworthy. 9 18 19 20 21 22 In particular, rapid clot formation is dependent on rapid generation of thrombin as well as fibrin generation and cross-linking, both parameters that can be altered by treatment with anticoagulants. 23 In addition, anticoagulants such as heparin or factor Xa antagonists loosen clot structure, rendering it more susceptible to fibrinolysis. 20 This may be important, since the recent “Cardiovascular Outcomes for People Using Anticoagulation Strategies” (COMPASS) trial demonstrated superiority of combined treatment with low-dose rivaroxaban and aspirin over aspirin alone in the prevention of recurrent events in patients with stable CAD. 5 The combination of rivaroxaban 2.5 mg twice daily and aspirin 100 mg daily demonstrated a reduction in combined cardiovascular ischemic endpoints, and also overall mortality as compared with aspirin alone. 5 The protection from ischemic events in COMPASS came at the cost of a mild increase in bleeding events, which may limit the broad applicability of combined aspirin and rivaroxaban in clinical practice. Thus, an ex vivo assay that may identify subjects with evidence of procoagulant tendency, such as low K and high MA, may possibly be able to identify patients who most benefit from treatment with anticoagulants.

The results of our study suggest that FXIIIa contributes at least in part to high clot strength phenotype, and may in part contribute to risk of subsequent coronary thrombotic events, although only modestly. The importance of FXIIIa in both venous and arterial thrombosis is increasingly recognized. 13 The majority of circulating FXIII is stored in platelets, and FXIIIa exposed on the surface of activated platelets contributes to stabilization of FXIII-depleted thrombi and antifibrinolytic function. 13 24 25 Red blood cell retention in whole blood thrombus is dependent on FXIIIa. 26 Platelets from FXIII-deficient patients exhibit decreased platelet activation and adhesion to fibrinogen. 27 We have previously demonstrated a rise in FXIIIa concentration in plasma supernatant occurring after platelet aggregation, suggesting some degree of release of FXIIIa into plasma from platelets, the mechanism of which remains unclear. 28 It is reasonable to assume that platelets activated at the site of arterial thrombus contribute to fibrin clot stabilization either by direct or indirect exposure of FXIIIa contents. In our study, we may have underestimated the effect of platelet-bound FXIII by utilizing platelet-poor plasma for TEG measurements and FXIIIa assays. The contribution of platelet-bound FXIIIa may be detected with whole blood–based TEG assays, but the differential contribution of FXIIIa activity versus platelets and fibrin is more difficult to interpret. While similar in concept, the measurements of plasma fibrin MA in our study are higher than what is generally observed for activator channel used in whole blood TEG platelet mapping systems. Activator channel in whole blood TEG platelet mapping assay uses externally added reptilase to cleave fibrinogen into fibrin in the absence of thrombin formation which is inhibited by heparin, as well as added FXIIIa to cross-link fibrin, thus not requiring in vivo cleaving of FXIII by thrombin into A and B subunits. The difference in MA between the methods is consistent with lower clot strength seen for same TEG assays performed in whole blood as compared with platelet-rich plasma, possibly due to incorporation of red cells into fibrin network. 1 Also the difference in anticoagulants in both assays (citrate vs. heparin) and absence of thrombin in activator channel could influence effectiveness of fibrin cross-linking and the final result of fibrin clot strength. Due to extrinsic addition of FXIIIa in activator channel of whole blood TEG assays, activation of FXIII does not occur at sites usually exposed to thrombin, such as platelets, microparticles, or red cells. Incorporation of red cells into whole blood thrombus has been shown to be dependent on FXIIIa adding resistance to fibrinolysis. 13 26 Differences between other functional fibrinogen assays and plasma fibrin clot strength measured in platelet-poor plasma have been previously reported for ROTEM assay. 29

While the adjusted hazard ratios for high TEG-MA, low TEG-K, and high FXIIIa are relatively modest, they are still higher than relative risk observed for recurrent MACE with high PRU measured by VerifyNow P2Y12, a now well-accepted measure of high on treatment platelet reactivity and risk variable after coronary stenting. 30

Given the associated bleeding risk with any form of intensified antithrombotic treatment regimens, in particular in subjects at higher risk for bleeding in general, the utility of an ex vivo assay that possess predictive qualities for both risk and harm increases. This is a role that TEG as global thrombosis assay may be poised to accomplish.

Further investigations to assess the utility of inhibition of FXIIIa to moderate thrombotic risk may be warranted; however, this therapeutic approach may be limited by the increased risk of adverse bleeding as observed in patients with inherited FXIII deficiency. 31

Limitations of our study include the relative small sample size and the relatively modest predictive values for TEG-MA, TEG-K, and FXIIIa. Our study was not powered to evaluate the performance of TEG or FXIIIa activity measurements in prediction of bleeding risk, and we did not include fibrinogen measurements. In addition, the contribution of FXIIIa stored in platelets to thrombosis may have been underestimated by use of platelet-poor plasma samples in TEG measurements.

## Conclusion

High plasma fibrin clot strength (TEG-MA), short clot formation time (TEG-K), and high FXIIIa were associated with increased risk of recurrent thrombotic events after coronary intervention. High plasma fibrin clot strength (TEG-MA) without incorporation of other parameters was superior to combination of all parameters in combined predictive risk scores.
